# Preclinical Alzheimer’s dementia: a useful concept or another dead end?

**DOI:** 10.1007/s10433-022-00735-w

**Published:** 2022-10-19

**Authors:** Ruth E. Mark, Yvonne Brehmer

**Affiliations:** 1 Department of Cognitive Neuropsychology, Tilburg University, PO Box 90153, 5000 LE Tilburg, The Netherlands; 2 Department of Developmental Psychology, Tilburg University, PO Box 90153, 5000 LE Tilburg, The Netherlands; 3 Aging Research Center, Karolinska Institutet, Stockholm, Sweden

**Keywords:** Alzheimer’s dementia, Biomarkers, Ethics, Mild cognitive impairment, Preclinical (Alzheimer’s) dementia

## Abstract

The term, *preclinical dementia,* was introduced in 2011 when new guidelines for the diagnosis of Alzheimer’s dementia (AD) were published. In the intervening 11 years, many studies have appeared in the literature focusing on this early stage. A search conducted in English on Google Scholar on 06.23.2022 using the term “preclinical (Alzheimer’s) dementia” produced 121, 000 results. However, the label is arguably more relevant for research purposes, and it is possible that the knowledge gained may lead to a cure for AD. The term has not been widely adopted by clinical practitioners. Furthermore, it is still not possible to predict who, after a diagnosis of preclinical dementia, will go on to develop AD, and if so, what the risk factors (modifiable and non-modifiable) might be. This *Review/Theoretical* article will focus on preclinical Alzheimer’s dementia (hereafter called preclinical AD). We outline how preclinical AD is currently defined, explain how it is diagnosed and explore why this is problematic at a number of different levels. We also ask the question: Is the concept ‘preclinical AD’ useful in clinical practice or is it just another dead end in the Holy Grail to find a treatment for AD? Specific recommendations for research and clinical practice are provided.

## Introduction

A revision of the old NINCDS‐ADRDA Work Group guidelines (McKhann et al. 1984) for the diagnosis of Alzheimer’s dementia (AD) appeared in 2011 (Sperling et al. 2011). The most important change was the recognition that AD progressed along a continuum. Two new stages were also introduced, namely the preclinical stage, traditionally seen as a “symptom-free” period of 10–20 years before the actual diagnosis was given followed by a prodromal period labeled ‘Mild Cognitive Impairment’ (MCI). MCI was said to occur when cognitive impairment was worse than expected for the person’s age but not enough to be classified as dementia and where daily functioning was not impaired.

Furthermore, while the old guidelines focused on memory loss as the first sign of impending AD, researchers now recognized that other cognitive impairment (e.g., judgment, planning, word finding) could occur before (or instead of) memory problems. The new guidelines therefore recognized the importance of the long, gradual progression of AD and the different transitional phases before an actual clinical diagnosis was made. In addition, for the first time, the role biomarkers could play in (early) diagnosis was introduced. These biomarkers (e.g., peptide amyloid-beta A*β* (A*β*+)—see paragraph on biomarkers below) were purported to suggest underlying disease before any clinical symptoms were obvious/noticeable (Jack et al. 2018). Biomarkers could also be employed to give more insights into the type and possibly also the cause of disease. Indeed, many researchers now state that AD is a biological disease (Jack et al. 2018) with specific features and that these can be identified before (or instead of) the need for clinical symptoms. Not everyone agrees however with this medicalization of AD (see for example McCleery et al. 2019). Furthermore, the term preclinical AD has not been widely accepted in or recommended for clinical practice (Brooker et al. 2014) making its practical use questionable at best.

### What is the preclinical stage?

This stage has been estimated to begin between 10 and 20 years before a dementia diagnosis and where clinical symptoms are not yet present/overt (Sperling et al. 2011). It is also of course possible that the tests traditionally used to differentiate and diagnose AD are not sensitive or specific enough to detect subtle cognitive changes early in the continuum. One-off (cross-sectional) measurements may also obscure (or not even detect) subtle, gradually changing cognitive decline, especially in the early stages.

Given that age is the number one risk factor for late-onset AD, or indeed most neurodegenerative diseases (Hou et al. 2019), this suggests that, for most people, the preclinical stage will begin in or before midlife (around 40–60 years of age). The question is however how can we know that someone is in the preclinical stage when clinical/overt symptoms are not present? Studies have shown that impairment in the brain occurs before these symptoms appear. Typically, abnormal levels of peptide amyloid-beta A*β* (A*β*+) in the cerebrospinal fluid or assessed via positron emission tomography (PET), in clinically normal (CN) individuals, are taken as a sign of preclinical AD (Jack et al. 2018; Sperling et al. 2011). The first affected brain regions include the entorhinal cortex, which is followed by the hippocampus, the rest of the medial temporal lobe and, as time progresses, deterioration spreads to the rest of the brain including the frontal regions (but see Tenenholz Grinberg (2017) who purports that *subcortical* structures are first affected by accumulation of Tau including the locus coeruleus). Recent studies also suggest that different subfields within the hippocampus not only decline at different rates but are also linked to changes in both objective and subjective memory measures (e.g., see Cremona et al. 2021 among others).

### Biomarkers for AD

The amyloid cascade hypothesis (Hardy and Higgins 1992) has long held sway in the field of AD research and suggests that there is a buildup of the peptide amyloid-beta (A*β*) in the brains of people with AD. At some point, this will lead to the extracellular formation of plaques, while accumulation of tau protein(s) has been linked with intracellular tangle formation (Bloom 2014). Both ultimately cause neuronal cell death and mark the onset of AD. There are both proponents and opponents to this hypothesis. The biggest problem is that healthy, older people can also have not only amyloid buildup but also tangles and plaques in their brains. Drug trials, which traditionally focus on removing amyloid or preventing it from forming, have also spectacularly failed to date (Yiannopoulou et al. 2019). The debate as to what causes AD rages on and this “Alzheimer conundrum” (why people whose brains show amyloid buildup, tangles and plaques do not always ‘convert’ to AD) is nicely argued in Margaret Lock’s popular book (Lock 2013). For a critical review on the amyloid cascade hypothesis, see Reitz 2012. Dubois et al. (2016) sum this conundrum up as follows, quote: “a sizable minority of cognitively normal older individuals will die with a high-amyloid burden but without experiencing discernable cognitive impairment during life” (p. 14). How these deposits develop has been staged in vivo (i.e., amyloid sensitive PET imaging in living individuals; Grothe et al. 2017) but what triggers them and what tips someone over into symptomatology severe enough to warrant an AD diagnosis is not yet known.

Current work on biomarkers for AD focuses on various types of Tau, Amyloid, plasma neurofilament light protein (NfL) (Rafii 2018), and genetic markers including the ε4 allele of the apolipoprotein E (APOE) gene, presenilin 1 and 2 (PSEN1, PSEN2) and many more (at least 20 loci were named by Giri et al. 2016, in late-onset AD and this number continues to rise). None of these are specific to AD, for example plasma NfL is age-linked and a marker for neurodegeneration (levels are enhanced in other brain disorders including Parkinson’s disease, Vascular dementia and others; Jin et al. 2019).

There are currently four different classification systems (employed mainly by researchers) to identify preclinical AD, all of which are based on biomarkers. These four classifications include A/T/N—the only one which deciphers phosphorylated or *p*-Tau and total or *t*-Tau (Jack et al. 2016); Dubois criteria (Dubois et al. 2016); International Working Group-2 (IWG-2) criteria (Dubois et al. 2014); and the National Institute on Aging–Alzheimer’s Association (NIA-AA) Criteria (Sperling et al. 2011; Jack et al. 2012). For details (beyond the scope of this paper) on how to use these classifications systems, see Kern et al. (2018).

### The problems with biomarkers

While biomarkers are increasingly utilized to identify preclinical disease (Lu et al. 2019), a major difficulty remains. That is, biomarkers are not routinely measured in either community populations or even in routine clinical testing as they are expensive and the means and/or expertise needed to measure them are not always available. They are therefore currently limited to (experimental) laboratory settings and/or select populations (e.g., they are sometimes measured in people who are said to be ‘high-risk’ because AD runs in their families). Biomarker findings are also notoriously difficult to replicate over different laboratories and should therefore not be used as a definite diagnostic criterion (Shi et al. 2018). At best, they can support a diagnosis. Ideally, a simple blood test conducted in community settings, which identifies what the cutoff(s) should be to determine whether a person is in the preclinical stage of AD, could be the solution. While efforts are being made to develop such a blood test, it is not yet available but may be in the near future (Schindler et al. 2019). This does not stop advertisements for “genetic/blood” testing for AD popping up all over the Internet duping the general public into misclassification, stress and needless costs. They also feed into the anxiety many older people have of developing AD, the so called dementia worry (Kessler et al. 2012).

To be fair, the original developers of the A/T/N classification system stated that it “should not be used in general medical practice as it is premature and inappropriate.” Frisoni et al. (2019, p. 919) go further by saying that we should “refrain from using the Alzheimer’s disease label for cognitively intact people showing abnormal amyloid markers (CSF or PET) but normal or unknown tau markers.” They call this instead “*amyloidosis of the brain,*” and see this as “*a risk marker for neurodegenerative dementia.*”

The final problem with biomarkers is that none of them can fully predict the natural course of dementia even at the group level (Shi et al. 2018). However, a recent systematic review by Parnetti et al. (2019) found that, in the 36 included articles where biomarkers were assessed, the overall estimated prevalence of preclinical AD was 22%. These authors furthermore reported that (*p*. 1) “The risk of progression increases across preclinical AD stages, with individuals classified as NIA-AA Stage 3 showing the highest risk (73%, 95% CI = 40–92%) compared to those in Stage 2 (38%, 95% CI = 21–59%) and Stage 1 (20%, 95% CI = 10–34%).” Individual differences are rife in this population both in terms of when the diagnosis is given and the speed of deterioration over time.

### Heterogeneity of the AD population

There is no “gold standard” for a definite diagnosis of AD never mind the *preclinical* stage. Indeed, it remains shocking that the numbers of people with undetected dementia (i.e., those that remain undiagnosed in their lifetimes) are so high; Lang et al. (2017) quoted a pooled global estimate of 61.7%, while estimates have been as high as 90% in community samples in China (Chen et al. 2013). Lang et al. (2017) also stated that men and younger people were the most underdiagnosed, that there were fewer diagnoses in the community than in residential settings (the latter were still high at 50% not diagnosed), and that general practitioners do not always recognize the signs and symptoms of dementia onset (Ahmad et al. 2010), attributing them to normal aging.

Individual differences (in onset, presentation and progression) have typically been explained by the catch-all term “cognitive reserve” which has been defined as, quote: “…the adaptability (i.e., efficiency, capacity, flexibility … of cognitive processes that helps to explain differential susceptibility of cognitive abilities or day-to-day function to brain aging, pathology, or insult” (Stern et al. 2020; p.1306). The term was first introduced by Stern in 2002 as an attempt to explain why a direct relationship between the degree of brain pathology was not always observable in clinical symptoms or signs. Stern et al. (2020) also conceptualized brain reserve as (*p*. 1308): “Brain reserve (BR) is commonly conceived as neurobiological capital (numbers of neurons, synapses). BR implies that individual variation in the structural characteristics of the brain allows some people to better cope with brain aging and pathology than others before clinical or cognitive changes emerge.” Understanding why some individuals rapidly deteriorate while others stay stable or reverse to near normal is a puzzle that researchers are attempting to untangle. There is every reason to expect that this heterogeneity in AD is also apparent at the preclinical stage. This field of study is very much in its infancy hampered by the fact that biomarkers are not widely available or even able to determine if or when an individual will develop AD. Alexopoulos and Kurz (2015, *p.* 365) stated that “new AD diagnostic guidelines exaggerate the role of biomarker abnormality and overlook the multifactorial genesis of the disease.” These authors also called for further refinement of the criteria. As such then it has not been adopted (or indeed recommended; Brooker et al. 2014) in general clinical practice.

### Is there another way (besides biomarkers) to decipher if an individual is in the preclinical stage?

A cost-effective, simple, short, online cognitive test, which could be conducted in any setting, would be the ideal solution. Unfortunately, no such screen or test currently exists which has a high enough sensitivity and specificity to accurately diagnose preclinical AD. The most well-known cognitive screen, the Mini Mental State Examination (MMSE) (Folstein et al. 1975), was not designed to detect dementia and cannot make a differential diagnosis and cannot detect early, (pre-symptomatic) stages. At most it can gauge severity of decline as the AD progresses to the later stages (see Nieuwenhuis-Mark 2010, for a critique of the MMSE). However, work in this area is progressing rapidly (e.g., see AD Protect and Prevent tool; Ashford et al. 2019; Öhman et al. 2021). The Preclinical Alzheimer Cognitive Composite (ADCS-PACC) has, for example, been suggested as a potential candidate sensitive enough to detect preclinical AD (Donohue et al. 2014) but this work is still in the early stages and needs to be more thoroughly tested in community populations using longitudinal, prospective designs. Comparison of newly developed tests should, of course, be compared to those currently used in clinical practice and to existing datasets. If these new tests are also computerized, we will need to consider the advantages and disadvantages of such tests (beyond the scope of this paper but see Woo (2008) for a review on these issues). López-Sanz et al. (2019) put this succinctly when they stated that “our ability to distinguish at-risk individuals in the earliest stages of the disease is still relatively limited. Some at-risk conditions have been described at the individual level; yet reliable markers to classify subjects on an individual basis remain still relatively unknown” (*p*. 2).

### Is there evidence of effective treatment(s) for preclinical AD?

Drug trials, which are effective in changing the course of or indeed preventing AD from occurring in the first place, have been spectacular in their failure to date (e.g., Schneider et al. 2014). The hype surrounding the new drug Aducanumab, while potentially promising, awaits further testing, is unlikely to be suitable for all and also remains prohibitively expensive (see Knopman et al. 2020 for a critique). There is however some evidence (Baumgart et al. 2015) that there are modifiable factors, which, if targeted, could either prolong the person with AD’s life and/or enhance its quality. These include for example: management of cardiovascular risks factors (e.g., obesity, smoking, type 2 diabetes, hypertension), a healthy diet, and regular physical activity. This has been found to be more effective when modification/management of these factors begins in midlife (vs older age) (e.g., Malik et al. 2021; Lisko et al. 2021). It is also generally accepted, at least in the research community, that if we are to succeed at slowing down or even reversing AD, we need to begin early (midlife) before brain damage has become too severe. Learning over the lifespan and training cognitive functions may also be beneficial. However, cognitive training does not always result in what researchers call *transfer* (i.e., training specific cognitive functions which then generalize to other cognitive functions or everyday life activities) making the investment in such trainings disputable (e.g., Stojanoski et al. 2018). Reiman et al. (2016) provided a review of current treatment trials. Which of these modifiable factors and what combinations and/or intensity levels work are not yet known at the group never mind the individual level. In summary, there is no cure currently on the market which can prevent AD. Furthermore, even if a treatment does become available in the foreseeable future, it is unlikely to work equally for all individuals affected.

### The ethics of early diagnosis

The term *preclinical AD* focuses on a time in peoples’ lives when they are functioning normally, while their brains are slowly deteriorating. The need to diagnose as early as possible is based on the assumption that if we tackle (stop or slow down) the deterioration in the brain before it tips into AD then it would not only help the individuals affected to live impairment-free for longer but that it would also save the world’s economies from the enormous costs AD creates now and in the future. However, the question must be raised: Is it ethical to intrude in people’s lives when they are, from the outside at least, functioning normally? Who is benefitting from early diagnosis and what potential harm could it cause? The (potential) patient may want to know (or not) and their needs and those of their partners should always come first in any clinical decision.

Launer (2019) also alerts researchers to the fact that there is still no standard way of assessing AD, while Langa and Burke (2019) highlight the risk of overdiagnosis and subsequently higher costs than is currently the case in health care for older individuals. A worrying trend was found for example in an MCI population who had *negative* PET scans for amyloid and 24% still continued to take drugs prescribed for AD (none of which have proven effective in stopping the disease or even in slowing it down) (Rabinovici et al. 2019). We simply do not know at this point whether anti-amyloid treatment at the preclinical or MCI stage can actually reduce the risk of later-onset AD (Karlawish and Langa 2016). Ideally, a risk estimate of who will convert (from preclinical AD and/or MCI to AD) is necessary in order to focus on those who could benefit most from early treatment. There is some recent research, which is attempting to do just this (see for example Jang et al. 2017; Payton et al. 2022).

It was recently estimated that 30% of the US population older than 50 years had increased amyloidosis but no cognitive impairment (Brookmeyer et al. 2018) and were therefore liable to be given the label *preclinical AD.* Treating all who have preclinical AD with no real idea of how many will convert to AD (some may die from other causes before they do develop the disease) would simply be unsustainable (costs, resources) even if it was possible. This of course raises the questions: When is it too late? Is there a ‘point of no return’? Can different treatments be effective at different stages? We have no definite answers to any of these questions. Costs of AD and other dementias are currently sky high and set to get even higher: In 2015, these costs were estimated to be $818billion US dollars globally (Livingston et al. 2017). Of course, costs cannot only be measured in monetary terms but also in the health (mental, physical) of both the person diagnosed, in their immediate family members and in terms of social emancipation. Overcoming these (and other) challenges will be necessary to advance not only the research into effective treatments of AD but also underline whether preclinical diagnosis is useful in all situations/for all individuals (see Molinuevo et al. 2016 and Whitehouse 2019 for more detailed critiques of the ethical issues surrounding preclinical AD.)

### Specific recommendations for research and clinical practice

The term preclinical AD is currently only used for research purposes. Whether it should stay this way is questioned in the current article. It has great clinical relevance in that if detected early enough the people who are diagnosed with it could be targeted with specific interventions to slow down the AD disease process and perhaps even reverse it. Of course, there are people who will want to know and others who will not. This should be their personal choice. The point is, they should be given that choice. Currently, these people will be diagnosed too late to make any difference to their disease progression. Which specific interventions could work for these individuals in the preclinical phase are as yet unknown.

For preclinical AD to become a useful clinical diagnosis, a few things would need to happen. First, detection would have to become easier and more cost-effective. Distinguishing people with preclinical AD from normal aging could feasibly be done if a sensitive enough cognitive test was available. Taking individual differences into account (e.g., interests, lifestyle, coping, mood, social context), rather than relying only on group norms, could be the way forward here. A comparison with what is already available (e.g., neuropsychological tests, biomarkers if available, existing datasets) in normal (people without disease) and clinical populations (people with subjective cognitive decline, MCI, early stage AD) would be needed. Some attempts are currently being made to explore this complex field. More emphasis on the individual(s) concerned, and their needs and wishes are recommended using a holistic approach (see for example Davitt et al. 2016) that focuses on much more than simply underlying brain abnormalities.

## Conclusions

The term *preclinical AD* is now mainstream in aging research. It typically refers to the first stage in the continuum from healthy/normal aging toward AD. While widely accepted, at least in scientific research circles, it remains difficult to both diagnose and predict progression in the individual patient. Biomarkers are currently a requirement for the diagnosis of preclinical AD, but they remain expensive and not available for all. Cost-effective cognitive screens/tests may be the way forward, while it remains crucial to be aware of the ethical quagmire of diagnosing people with a disease before symptoms manifest and where no cure exists. Understanding what occurs (cognitively, neurologically, functionally and emotionally) when individuals age is essential. Determining who is at risk and which factors could be protective and which heighten the risk of AD is a good development. Patients tend to be cared for by their partner or adult child(ren) and many remain at home for years before they are placed in an institution outside the home (Mark 2015). New biomarkers (Jongsiriyanyong and Limpawattana 2018) and/or prediction models (Khan 2018; Hall et al. 2019) incorporating a variety of relevant (sociodemographic, clinical, neurological and psychological) variables are being developed. They will hopefully not only highlight which individuals are at the highest risk for both developing AD and/or for rapid decline once diagnosed but will also guide future treatments and ultimately lead to a cure (i.e., stop dementia developing in the first place) or at least slow down its progression.

It is also possible that there is too much emphasis on the medicalization of AD and more research is needed, taking the individual, caregiver and the context into account (in other words, a ‘systems’ or holistic approach). Remembering the individual behind the label is paramount. Questioning and exploring the usefulness of the term *preclinical AD* and how and if it will progress could provide the clues needed for effective, person-centered treatments in the devastating diseases clustered under the term dementia. The economic sustainability of our aging world and the well-being and independence of older individuals will depend on finding effective treatments in the years ahead. Focusing research on preclinical AD, especially on discovering who is at risk for future development of AD and targeting modifiable factors, could provide new insights into a disease that remains incurable.

Is preclinical AD a useful concept? In short, the answer to this question is yes, if it leads to effective treatment(s) which could prevent AD from developing at all, or, at the very least, slow down the process. The concept is however not useful if it overdiagnoses and stigmatizes people unnecessarily who will ultimately not go on to develop AD in their lifetimes. Taking individual differences in risk factors, context, disease type(s) and progression into account will be paramount. Furthermore, developing sensitive (cognitive) tests which can detect preclinical AD and target treatments ideally before brain damage becomes irreversible is likely to be the way forward when expensive biomarkers are not available to all. Preclinical AD remains a concept worth exploring in our quest to understand and ultimately reduce the devastating impact AD has on all affected.
